# Female offspring gestated in hypothyroxinemia and infected with human Metapneumovirus (hMPV) suffer a more severe infection and have a higher number of activated CD8^+^ T lymphocytes

**DOI:** 10.3389/fimmu.2022.966917

**Published:** 2022-09-08

**Authors:** Samanta C. Funes, Mariana Ríos, Ayleen Fernández-Fierro, Daniela Rivera-Pérez, Jorge A. Soto, José R. Valbuena, María J. Altamirano-Lagos, Felipe Gómez-Santander, Evelyn L. Jara, Pablo Zoroquiain, Juan C. Roa, Alexis M. Kalergis, Claudia A. Riedel

**Affiliations:** ^1^ Millennium Institute on Immunology and Immunotherapy, Departamento de Genética Molecular y Microbiología, Facultad de Ciencias Biológicas, Pontificia Universidad Catóica, de Chile, Santiago, Chile; ^2^ Instituto Multidisciplinario de Investigaciones Biológicas-San Luis (IMIBIO-SL), Consejo Nacional de Investigaciones Científicas y Técnicas (CONICET), Universidad Nacional de San Luis (UNSL), San Luis, Argentina; ^3^ Millennium Institute on Immunology and Immunotherapy, Departamento de Ciencias Biológicas, Facultad de Ciencias de la Vida, Universidad Andrés Bello, Santiago, Chile; ^4^ Departamento de Anatomía Patológica, Facultad de Medicina, Pontificia Universidad Católica de Chile, Santiago, Chile; ^5^ Departmento de Farmacología, Millennium Institute on Immunology and Immunotherapy, Facultad de Ciencias Biológicas, Universidad de Concepción, Concepción, Chile; ^6^ Departamento de Endocrinología, Escuela de Medicina, Facultad de Medicina, Pontificia Universidad Católica de Chile, Santiago, Chile

**Keywords:** thyroid hormones, gestational hypothyroxinemia, human metapneumovirus (hMPV), viral infection, metapneumo virus

## Abstract

Maternal thyroid hormones (THs) are essential for the appropriate development of the fetus and especially for the brain. Recently, some studies have shown that THs deficiency can also alter the immune system development of the progeny and their ability to mount an appropriate response against infectious agents. In this study, we evaluated whether adult mice gestated under hypothyroxinemia (Hpx) showed an altered immune response against infection with human metapneumovirus (hMPV). We observed that female mice gestated under Hpx showed higher clinical scores after seven days of hMPV infection. Besides, males gestated under Hpx have higher lung viral loads at day seven post-infection. Furthermore, the female offspring gestated in Hpx have already reduced the viral load at day seven and accordingly showed an increased proportion of activated (CD71^+^ and FasL^+^) CD8^+^ T cells in the lungs, which correlated with a trend for a higher histopathological clinical score. These results support that T_4_ deficiency during gestation might condition the offspring differently in males and females, enhancing their ability to respond to hMPV.

## Introduction

Maternal thyroid hormones (THs) 3,5,3¨-L-triiodothyronine (T_3_) and 3,5,3¨5¨-L-tetraiodothyronine (T_4_) are essential for the proper development of the fetus, especially the central nervous system (CNS), lungs, skeletal muscles and bones of mammals (1, 2). Thyroid hormones are tyrosine-based hormones secreted by the thyroid gland with metabolic functions; nevertheless, T_3_ is considered the biologically active form of the THs, whereas T_4_ is their precursor (3). Indeed, around 80% of the circulating T_3_ is produced out of T_4_ by the action of deiodinases, highlighting the importance of T_4_ production (4).

During the gestation period, the hypothalamic-hypophysis-thyroid axis of the fetus is immature, and consequently, the fetus depends on maternal THs that will ensure his/her proper development (5, 6). Mainly T_4_ crosses the placenta barrier, and the fetal tissues will transform T_4_ into T_3_ by the action of deiodinases (7, 8). THs deficit in pregnant women has been clearly associated with irreversible damage to the offspring CNS, such as low intellectual quotient, attention deficit, mental disability and cretinism (9, 10).

Hypothyroxinemia (Hpx), unlike hypothyroidism, is less understood in clinical medicine. One of the reasons is that in Hpx, only plasmatic levels of T4 are reduced, while T_3_ and thyroid-stimulating hormone (TSH) levels remain normal (11). Consequently, usually adult patients do not show significant symptoms, and this condition is not diagnosed. However, for the fetus, gestational Hpx implies an entirely different situation from adults because only T_4_ and not T_3_ can reach fetal tissues across the placental barrier (12). Although most of the research has been focused on the effect of Hpx on CNS development, only a few reports have studied the impact on immune system development (13–16). Some of these studies performed in different animal models evaluated THs deficiency´s influence on B-lymphopoiesis, thymus physiology, and both cell-mediated and humoral immune responses (13, 16). In this regard, it has been observed that mice gestated under THs deficiency suffered a more severe manifestation of Experimental Autoimmune Encephalomyelitis (EAE) during adulthood as compared to mice gestated in euthyroidism (Eut) condition (17, 18). Treg and T CD4^+^ lymphocytes derived from mice gestated in Hpx have a reduced capacity to suppress effector T cell proliferation and differentiate into Treg cells, respectively (18, 19). These mice also showed high basal levels of TNF-α and IL-17 and more Th17 cells (19). Moreover, female mice gestated under hypothyroidism had increased vascular permeability in the lungs and an augmented resistance to respiratory bacterial infections such as *Streptococcus pneumoniae* (20). Furthermore, these mice showed higher amounts of inflammatory cells in the lungs and reduced expression of IL-6 cytokine 48 hours after infection (20). The fact that mice gestated in THs-deficient condition are resistant to a respiratory bacterial infection prompted us to study whether gestational Hpx makes the offspring respond differently to challenges with a viral respiratory infection. This question was approached in the present work by evaluating the immune response induced by the human metapneumovirus (hMPV). This member of the *Pneumoviridae* family produces a large spectrum of illnesses in both upper and lower respiratory tracts (21). Besides, hMPV is considered one of the most prevalent viruses that cause young children’s hospitalization (22). In this study, we have examined the outcome of the infection with hMPV in adult male and female mice gestated in the Hpx condition. Indeed, we observed that animals who were gestated under the Hpx condition had a higher clinical score after seven days of infection.

Furthermore, females and males responded differently to the infection with hMPV. Here, we identify that female but not male mice gestated under Hpx achieved viral clearance at day seven after infection. Moreover, female progeny gestated in the Hpx condition displayed higher CD8^+^ T cell infiltration in the lungs, and these cells were highly activated, as evidenced by the expression of CD71 and FASL. In association, there is a tendency of increased lung inflammation, associated with a higher infiltration of activated CD8^+^ T lymphocytes compared with the control progeny gestated in euthyroid conditions.

## Material and methods

### Mice

BALB/cJ mice were purchased from The Jackson Laboratories (Bar Harbor, ME) and maintained under SPF conditions at the Pontificia Universidad Católica de Chile animal facility. All procedures were approved and supervised by the Scientific Ethics Committee for the Care of Animals and the Environment of the Pontificia Universidad Católica de Chile (reference number CBB-180411003). The protocol is under the Chilean Law 20380 on Animal Protection (2009), the Terrestrial Animal Health Code of the World Organization for Animal Health, the European Directive 2010/63/EU, and the Guide for the Care and Use of Experimental Animals.

### Induction of gestational Hpx in mice

Six-eight-week-old BALB/cJ mice were checked for vaginal plugs the day after the mating. Mice with vaginal plugs were considered pregnant, and that day was assigned as pregnancy day 1 (P1). From P10 to P15, mice were treated with 0.02% p/v methimazole (MMI) (M8506, Sigma-Aldrich, USA) (23) and 2% p/v of saccharose in the drinking water (Hpx group). On the other hand, in the control euthyroid group (Eut), the mice drank water without MMI during pregnancy. A second control group was included to restore euthyroidism. It consisted of pregnant mice that received 0,02% MMI in the drinking water and subcutaneous injections of 100 µl of T_4_ (25 µg/Kg body weight) from P10 to P15 (Hpx+T_4_ group). The experimental design is detailed in **Figure S1A**.

### Detection of thyroid hormones

THs of pregnant mice were measured on the last day of treatment (P15) from blood samples (200 µl) obtained from the submaxillary vein. In contrast, THs of their respective progeny were measured at seven-to eight-week-old at the moment of their euthanasia from a cardiac puncture (**Figure S1A**). According to the manufacturer’s instructions, total serum T_4_ (tT_4_) and T_3_ were measured by radioimmunoassay using a commercial kit (Coat-A-Count Siemens Healthcare Diagnostics kits, cat no. TKT41 and TKT31) in a specialized veterinary clinical laboratory. TSH was measured using a mouse Ultrasensitive Thyroid Stimulating Hormone (U-TSH) enzyme-linked immunosorbent assay kit (Mybiosource cat no MBS704901) according to the manufacturer´s instructions.

### Virus preparation and titration

LLC-MK2 (American Type Culture Collection, CCL-7TM) were used to propagate hMPV serogroup A, strain CZ0107 (clinical isolated and obtained from the Laboratorio de Infectología y Virología of the Hospital Clínico, Pontificia Universidad Católica de Chile) (24). Briefly, cell monolayers were grown in T75 flasks with DMEM (Life Technologies Invitrogen, Carlsbad, CA) supplemented with 10% FBS (Gibco Invitrogen Corp, Carlsbad) for HEp-2 cells and Opti-MEM supplemented with 5% FBS for LLC-MK2 cells until 80–90% confluence. Flasks containing 5 ml of infection medium (Opti-MEM 5% FBS medium, supplemented with 100 μg/ml CaCl_2_) were inoculated with 2×10^5^ Plaque formation units (PFU) of the respective virus and incubated at 37°C. After viral adsorption (2 h), supernatants were replaced with fresh medium (Opti-MEM) and incubated for 72 h until a visible cytopathic effect was observed. For harvesting, cells were scraped, and the flask content was pooled and centrifuged first at 300 × g for 10 min and then at 500 × g for 10 min to remove cell debris. In parallel, supernatants of non-infected LLC-MK2 were collected as previously described and used as non-infectious control (Mock). Viral titers of supernatants were determined by immunocytochemistry in 96-well plates with LLC-MK2 cells monolayers, as previously described (25). hMPV inoculums were routinely evaluated for lipopolysaccharide and Mycoplasma contamination. Both supernatants (hMPV and Mock) were stored at -80°C until the time of infection.

### Mouse challenge with hMPV

Six- to eight-weeks-old BALB/cJ progeny mice (three to five animals per group from three independent experiments) were anesthetized with a mixture of ketamine/xylazine (20 mg/kg and 1 mg/kg, respectively) and challenged by intranasal infection with 1 × 10^6^ PFU/ml of hMPV in a final volume of 100 μl per mouse. The body weight and clinical score (appearance and behavior) were recorded daily during the hMPV challenge (7 days after the infection), according to **Table 1**. All animals were manipulated with the supervision of a veterinarian and according to the institutional guidelines of the Pontificia Universidad Católica de Chile and the “Guide for the care and use of laboratory animals.”

**Table 1:** Variables considered in the clinical score.

Variables to consider	Observations	Score
Body weight	Normal.	0
	Loss of less than 10% of the initial weight.	1
	Body weight loss of between 10%-20% of the initial weight.	2
	Weight loss of greater than 20% of the initial weight.	3
Aspect	Normal, upright, shiny coat.	0
	Avoid moving, shaggy fur.	1
	Stooped, motionless, shaggy coat, porphyric secretion.	2
	prostrate, dehydration, sunken eyes.	3
Spontaneous behaviour	Normal, attentive to the environment, interaction, groomed.	0
	Small changes, reduced grooming and movement, rapid respiratory movements.	1
	Staggering movement, inactive or withdrawn, abdominal breathing.	2
	Immobile, prostrate, mouth open when breathing.	3

The table summarizes the parameters considered to elaborate the daily total score after hMPV infection.

### Sample collection

Mice were euthanized on the seventh day post-infection (dpi). Blood samples were collected at this point by cardiac puncture to measure tT_4_, T_3_ and TSH as described above. Next, the non-lobed section of the lung was clamped and stored for histological analysis (26).

### Determination of viral load by quantitative real-time PCR

The lower section of the lobate lung was collected for RNA purification. Total RNA was isolated from 100 mg lung tissue using TRIzol Reagent (Invitrogen, Carlsbad, CA.). According to the manufacturer’s instructions, 1 μg of RNA was reverse transcribed to cDNA using the iScript cDNA Synthesis Kit (BIO-RAD). Primers used for hMPV-N gene detection in quantitative real-time PCR (QPCR) reactions were Fwd: 5′- ACA GCA GAT TCT AAG AAA CTC AGG-3′ and Rev: 5′-TCT TTG TCT ATC TCT TCC ACC C-3′. Detection of mouse β-actin was used as a housekeeping reference gene, using the following primers Fwd: 5′-GGC TGT ATT CCC CTC CAT CG-3′ and Rev: 5′-CCA GTT GGT AAC AAT GCC ATG T-3′. The products were detected using Supermix SsoAdvanced Universal SYBR Green (BIO-RAD) in an Applied Biosystems StepOnePlus Real-Time PCR System Relative. Gene expression data analyses were performed using the comparative cycle threshold method. Standard curves for qPCR were generated by using pTOPO-N-hMPV or pTOPO-β-actin as templates. The C_T_ threshold results were entered in the standard curve with the quantity log. Viral load data were expressed as the number of copies of hMPV N-gene for 5000 copies of the β-actin transcript.

### Cytokines determination by qPCR

As was previously described, total RNA was isolated from lung tissue and reverse transcribed to cDNA. The abundance of IL-6, IL-10 and IFNγ mRNAs was determined by relative expression to the respective housekeeping gene. The following primers were used: mouse IL-6 Forward 5´- TAGTCCTTCCTACCCCAATTTCC-3´ and Reverse 5´-TTGGTGCTTAGCCACTCCTTC-3´, mouse IL-10 Forward 5´-GCTCTTACTGACTGGCAT-3´ and Reverse 5´-CGCAGCTCTAGGAGCATGTG-3´, mouse IFN-γ Forward 5´-ACAGCAAGGGGAAAAAAAGGATG-3´ and Reverse 5´-TGGTGGACCAGTCGGATGA-3´, mouse β-actin Forward 5´-ACCTTCTACAATGAGCTGCG-3´ and Reverse 5´-CTGGATGGCTACGTACATGG-3´.

### FACS analysis of lungs

The rest of the lung that was not used for RNA purification. Briefly, tissue samples were homogenized after 45 min incubation at 37 °C with collagenase IV (1 mg/ml) in sterile PBS. After that, lung homogenates were passed through a 70 μm cell-strainer, treated for red blood cells lysis, resuspended in PEB buffer (EDTA 2nM, BSA 0,5% in PBS), and stained with BD HorizonTM Fixable Viability Stain 700 following the manufacturer’s instructions. Consequently, samples were incubated with 0.2 mg/mL of BV510- anti-CD45 (Clone 2D1, BD Horizon), PE- anti-Ly6C (Clone AL-21, BD Pharmingen), Peridinin chlorophyll protein (PerCP) Cy5.5- anti-LY6G (Clone 1A8, BD Pharmingen), APC-Cy7- anti-CD11b (Clone M1/70, BD Pharmingen) and APCCy7-anti-CD8 (Clone SK1, BD Pharmingen), BV605- anti-IA/IE (Clone 25-9-17, BD OptiBuild), BV650- anti-CD4 (Clone L-200, BD Horizon), BV711- anti-CD3 (Clone 17A2, BD OptiBuild), BV786- anti-CD69 (Clone FN50, BD Horizon), PE-Cy7- anti-CD11c (clone HL3, BD Pharmingen) and PE-Cy7- anti-CD19 (Clone 1D3, BD Pharmingen), BUV395- anti-CD71 (Clone C2, BD OptiBuild), AF645- anti-CD64 (Clone X54-5/7.1, BD Pharmingen), PECF594- anti-Singlec-F (Clone E50-2440, BD Horizon) and BV421-conjugated to anti-CD178 (FasL) antibodies for 30 min at 4°. For GRZ B intracellular staining, fixed cells were incubated for 1 hr with a PerCP Cy5.5- conjugated to anti-GRZ B antibody in BD Cytofix/CytopermTM (BD Biosciences Pharmingen) permeabilization buffer (27). Cells were washed with permeabilization buffer and acquired using a BD LSRFortessa X-20 flow cytometer (BD Biosciences Pharmingen). To discriminate between CD45 single cells, doublets, and between live cells and cell debris, events were gated sequentially on side-scatter (SSC)-A and forward-scatter (FSC)-A, FSC-H and FSC-A, SSC-A and Alexa 700, and SSC-A and CD45-BV510 plots. Data were acquired in LSR Fortessa X20 (BD bioscience, USA) and analyzed using FlowJo v10.0.7 software (BD Biosciences). The gating strategy to identify the various cell populations is shown in the **Figure S2**.

### Anti-hMPV IgG antibodies determination

ELISA plates were coated with hMPV (previously UV-inactivated for 45 min and sonicated by 10 min to expose as many antigens as possible) overnight at 4°C, then blocked for one hour at room temperature and incubated for one hour with 100 μl of the different serum samples previously diluted at 1:500 in triplicate. Then, the plates were washed three times and incubated with 50 μl of 1:2,000 dilution of HRP-Goat anti-mouse IgG (H+L) (Life Technologies, N. Meridian rd., Rockford, IL 61101, USA) for one hour. Afterwards, plates were washed and revealed with 50 μl of 1 mg/ml 3-39-5-59-tetramethylbenzidine (TMB, Merck) in the darkness. After 10 min, 50 μl of H_2_SO_4_ solution was added to stop the reaction. Plates were analyzed in an ELISA reader at 450 nm (Multiskan Ex, Thermo Labsystems).

### Lung histopathology

Non-lobed sections of the lung were removed aseptically and fixed for at least 24 hours in 4% paraformaldehyde-PBS (Merck Millipore, Billerica, MA.), embedded in paraffin, cut with a microtome into five μm-thick sections, and adhered to slides. Hematoxylin and eosin staining were performed with Harris Hematoxylin (Leica biosystems, Wetzlar) and Aqueous Eosin Y (Thermo Fisher Scientific, Waltham.). The slides were then converted into digital images using the Aperio scanner AT2 (Aperio, Leica, Richmond, IL, USA). The scanned slide images were analyzed by a board-certified pathologist blinded to the animal’s status as per criteria modified from Cimolai et al., 1992 (28) based on the morphological findings hRSV from Johnson et al., 2007 (29). The score used was the following: 1) Peribronquial/bronchiolar infiltrate area: The area was analyzed semi-quantitatively as 0 when no infiltrate was observed, 1 when less than 50% of the structures had inflammation, and 2 when higher than 50% of the structures were inflamed. 2) Peribronquial/bronchiolar infiltrate density: The density was semi-quantitatively scored as 1 mild, 2 moderate, and 3 intense. 3) Perivascular infiltrate: the area was analyzed semi-quantitatively as 0 when no infiltrate was observed, 1 when less than 50% of the structures had inflammation, and 2 when higher than 50% of the structures were inflamed. 4) Perivascular infiltrates density: the density was semi-quantitatively scored as 1 mild, 2 moderate, and 3 intense. 5) Intrabronquial cells: presence of inflammatory cells in the lumen of bronchial/bronchiolar structures as 0 no cells seen, 1 only scattered inflammatory cells, 2 inflammatory cells and detachment of bronchial cells, and 3 syncytial epitheliums with denudation of the epithelium. 6) Alveolar infiltrate area: the intra-alveolar occupied area was semi-quantitatively scored as 0 when no inflammatory infiltrate was present and 1 when less than 33% was infiltrated, 2 between 33% and 66%, and 3 when higher than 66% was infiltrated. In the mouse lung, unlike the human lungs, the terminal bronchioles lined by non-ciliated Clara cells lead directly to the alveolar lung. Accordingly, we counted the number of terminal bronchioles structures of each lung tissue histological slide at 4X objective microscopic field. We looked for structural differences between adult mice gestate under Hpx condition and normal Eut mice with this approach.

### Statistical analyses

Data and statistical analyses were performed using PRISM 8 software (Graph Pad Software, Inc., San Diego, CA). Data were obtained from three independent experiments, and each group of progeny mice has between 3 and 8 mice. Data are presented as standard error media or individual values as appropriate. One-way or two-way analysis of variance tests and Tukey multiple comparisons tests were used. P-values * < 0.05, ** < 0.01, ***<0.001 and **** < 0.0001 were considered statistically significant.

## Results

### Mice gestated under Hpx have normal levels of T_4_

To induce gestational Hpx, pregnant mice received a transient administration of MMI (19, 30). A schematical representation of the induction of the Hpx model is shown in **Figure S1A**. To corroborate the effectiveness of MMI treatment to induce Hpx, tT_4,_ T_3_ and TSH were measured in serum as described in materials and methods. As reported (18), the pregnant mice treated with MMI had significantly reduced levels of tT_4_ in sera (mean value of 0.9 µg/dl) compared to control Eut mice (mean value of 3.4 µg/dl) (**Figure S1B**). In turn, the level of TSH (**Figure S1C**) and T3 (**Figure S1D**) in the serum from mothers was also determined, and no changes were detected after MMI administration. Our results indicate that MMI treatment induces Hpx in pregnant mice, which was reversed with the administration of T_4_ during the treatment with MMI. Importantly, the MMI treatment during gestation did not affect the thyroid function of the progeny in adulthood, given that tT_4_ levels were similar to control groups (**Figure S1E**).

### Male and female mice gestated under Hpx condition respond differently to hMPV infection

We studied the outcome of hMPV infection on adult mice gestated under the Hpx condition by comparison with Eut and Hpx+T4 gestated mice. For that, all groups were challenged intranasally with 1x10^6^ PFU of hMPV or mock, and the associated disease parameters were evaluated.

**Figure 1:**
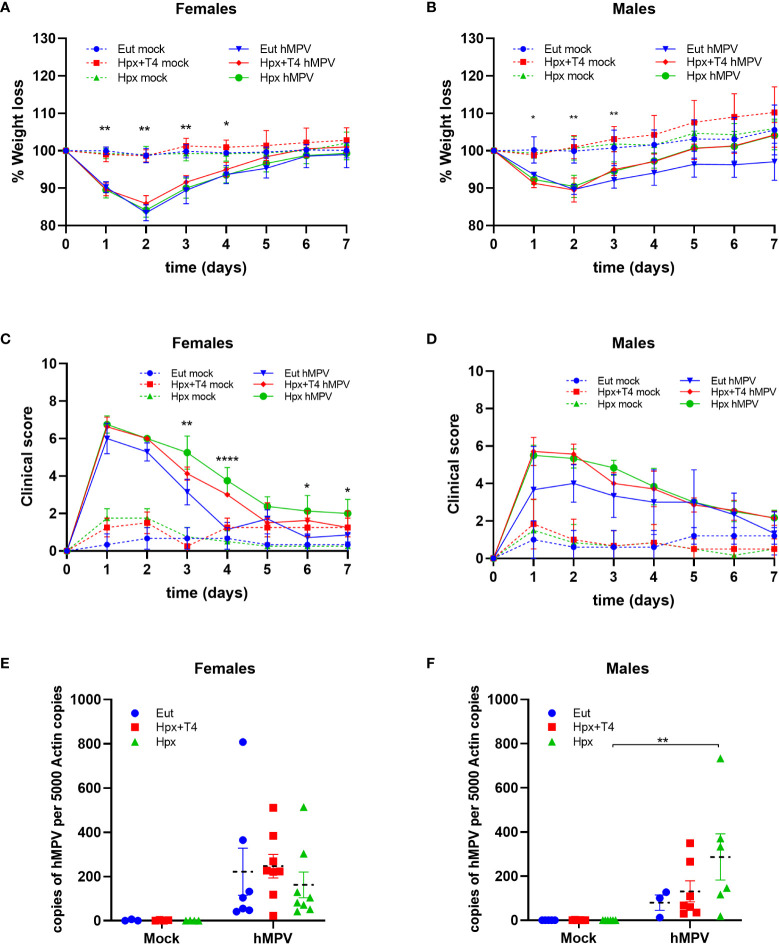
Male and female mice gestated under Hpx respond differently to hMPV infection. Mice from three experimental groups were infected with 1x10^6^ PFU of hMPV or mock. The graph shows the body weight loss of female **(A)** and male mice **(B)**, and a significant difference was evaluated between infected and non-infected animal groups. Mice were monitored seven days post-infection to record the clinical score, and the daily value for female mice **(C)** and male mice **(D)** was graphed. Statistical differences between Eut and Hpx groups are plotted. Viral loads were quantified as copies of the N-hMPV gene for female **(E)** and male mice **(F)**. Data are shown as the mean ± SEM of at least three animals per group. *p < 0.05 **p < 0.01 and ****p < 0:0001 by two-way ANOVA and Tukey’s multiple comparisons test.

All infected groups of female and male animals showed a significant weight loss compared with their respective mock control (**Figures 1A, B**). The maximum weight loss value was observed two days after the hMPV infection (14% in females and 10% in male animals). All hMPV-infected male groups recovered their initial weight on day four post-infection (**Figure 1A**). However, for the hMPV-infected female groups, the recovery of their initial weight was observed on day five post-infection, showing similar recovery kinetics between the different groups (**Figure 1B**). On day one post-infection, peak clinical scores were found for all infected animals (**Figures 1C, D**). Interestingly, in female mice, the hMPV Eut groups had a lower clinical score than the hMPV Hpx groups during 3-7 days after infection (**Figure 1C**). This difference was not observed in infected male groups (**Figure 1D**). Further, we evaluated all group´s viral lung loads on day seven post-infection (**Figures 1E, F**). Since that day, most of the hMPV infection has already been resolved (31); accordingly, viral load from infected female groups had no significant differences compared to mock groups (**Figure 1E**). Instead, Hpx male mice still showed significantly higher viral loads on day 7 (**Figure 1F**).

### Lungs of female mice gestated in hypothyroxinemia showed increased CD8^+^ T cells infiltration after hMPV infection

**Figure 2:**
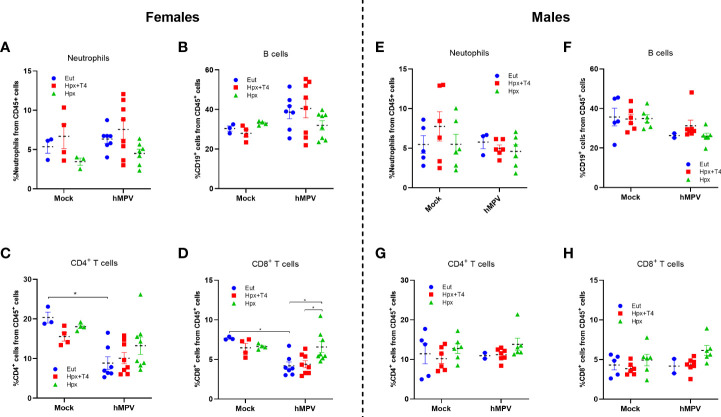
The hMPV infection increased the proportion of CD8+ T cells in the female Hpx progeny. Flow cytometry of single-cell suspension on CD45^+^ cells from lung seven days post-infection with 1x10^6^ PFU hMPV or mock were performed for Eut, Hpx+T4 and Hpx gestated mice. **(A, E)** Neutrophils (CD11c^-^ CD11b^+^ Ly-6G^+^ SIGLEC-F^-^), **(B, F)** B cells (CD3^-^ CD19^+^), **(C, G)** CD4^+^ T cells and **(D, H)** CD8^+^ T cells were analyzed for female **(A–D)** and male **(F–H)** mice. Data are shown as the mean ± SEM of at least three animals per group. *p < 0,05 by two-way ANOVA and Tukey’s multiple comparisons test.

Neutrophils, B lymphocytes, CD4^+^ and CD8^+^ T lymphocytes were quantified in the lungs of female and male mice of each experimental group by flow cytometry to address the cellular immune response after hMPV infection. There were no significant changes in the percentage of neutrophils in all groups of mice (**Figures 2A, E**). However, a trend for lower neutrophil infiltration was observed in hMPV-infected Hpx female mice compared to Eut and Hpx+T4 infected mice at seven dpi (**Figure 2A** **).** Interestingly, a tendency to decrease in the neutrophils was observed in hMPV infected male groups compared to mock-treated mice (**Figure 2E** **).** No significant differences were found in the percentage of B lymphocytes among all experimental groups (**Figures 2B, F**). Accordingly, no differences in anti-hMPV antibodies in the sera were detected (**Figure S3**). However, a tendency to increase the B cell population was observed from hMPV infected female groups compared to mock-treated mice (**Figure 2B**). Females Eut mock-treated showed a significantly high percentage of CD4^+^ T lymphocytes in the lung compared to females gestated in Eut at day seven after infection with hMPV. However, females gestated in Hpx retained a high percentage of these cells in the lungs after the hMPV infection (**Figures 2C**). For male groups, no significant differences between the groups were observed (**Figure 2G**). A significant increase in the percentage of CD8^+^ T lymphocytes infiltrating the lung was found in female mice gestated in Hpx and infected compared to Eut and Hpx+T4 female groups (**Figure 2D**). Male gestated under Hpx hMPV infected or mock-treated showed a tendency to increase the percentage of CD8^+^ T lymphocytes in the lung, but it was not significantly different (**Figure 2H**). Finally, the pulmonary infiltrate of other myeloid populations was also evaluated in each treated group without detecting significant differences between the animals gestated under different conditions (**Figure S4**).

### Increased CD8^+^ T cells with activated profile in female mice gestated in Hpx and infected with hMPV

**Figure 3:**
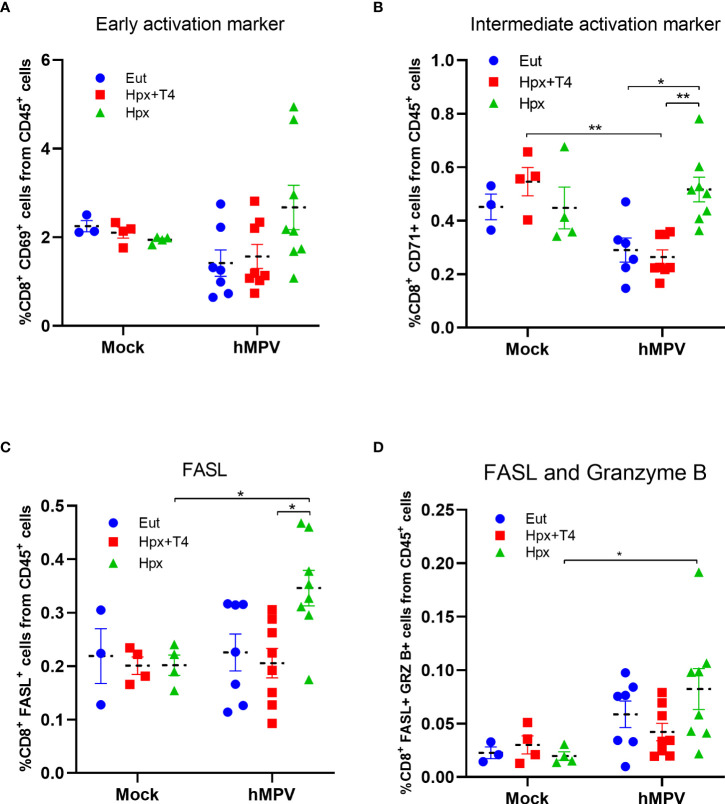
Female Hpx offspring present a high proportion of activated CD8+ T cells. Flow cytometry of single-cell suspension of CD8^+^ T cells on CD45^+^ cells from lung seven days post-infection with 1x10^6^ PFU hMPV or mock for each animal group. Percentage of cells expressing the activation markers CD69^+^ **(A)**, CD71^+^ **(B)**, FASL^+^ **(C)** and FASL^+^ GRZB^+^ **(D)** were analyzed in female mice from Eut, Hpx+T4 and Hpx mice. *p < 0.05; **p < 0.01 by two-way ANOVA and Sidak’ s multiple comparisons test. Data are shown as the mean ± SEM of at least five animals per group. SEM, standard error of the mean.

CD8^+^ T cells have a critical role in antiviral immune responses. Accordingly, the high percentage of CD8^+^ T lymphocytes in the lung of the female offspring gestated in Hpx could suggest a better antiviral immune response than males. Even though the proportion of CD8^+^ T lymphocytes expressing the early activation marker CD69 in females was similar between all experimental groups, a trend toward an increase in the Hpx female group was observed (**Figure 3A**). The proportion of CD8^+^ T lymphocytes with intermediate activation marker CD71 was significantly increased in the Hpx group compared to Eut and Hpx+T4 infected groups (**Figure 3B**). In contrast, the proportion of CD8^+^CD71^+^ T lymphocytes from the CD45^+^ population decreased in the lung of Eut and Hpx+T4 after the infection (**Figure 3B**). Fas ligand (FasL) and Granzyme B (GRZ B) as markers for effector function activity were evaluated on the CD8^+^ T lymphocyte population from CD45^+^ cells. Female Hpx infected mice showed a significant increase in CD8^+^FasL^+^ T lymphocytes than Hpx+T4 infected or Hpx mock-tread groups (**Figure 3C**). However, when the GRZB measurement was added to the FasL^+^ CD8^+^ T cell population (FasL^+^GRZB^+^CD8^+^), we found that significant differences were only maintained between the Hpx hMPV infected or mock-treated groups (**Figure 3D**).

### Lungs of mice gestated by Hpx mothers present a tendency of higher pulmonary inflammation after hMPV infection

**Figure 4:**
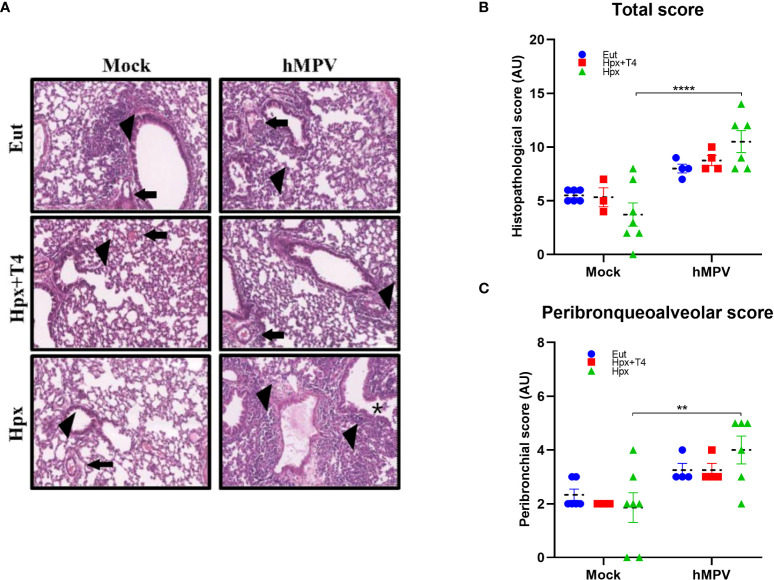
Hpx progeny tends to have a higher histopathological score after the hMPV infection. Lung sections from Eut, Hpx+T4, and Hpx male and female mice mock or hMPV infected are shown at 40X with H&E stain. A board-certified pathologist analyzed the degree of pulmonary inflammation for scanned slide images from each group. **(A)** Representative images of histological sections (600 µm) are shown. Arrowheads show peribronchial/bronchiolar infiltrate; arrows show perivascular infiltrates, and the asterisk indicates intrabronchial cells. **(B)** Histopathological score graph. **(C)** Peribronchiolar score graph. Comparisons for the same group were made using a two-way ANOVA and Tukey’s multiple comparisons test **p < 0.01 and ****p < 0.0001.

Histopathological analyses of the lungs of all experimental groups infected or not with hMPV were performed using the modified score from Cimolai et al., 1992 (28) and Johnson et al., 2007 (29). Lungs from Eut, Hpx, and Hpx+T4 mice from both sexes infected intranasally with hMPV had more pulmonary inflammation than mice treated with mock; representative histological processes are shown in **Figure 4A**. As expected, Hpx mice tend to increase pulmonary inflammatory cell infiltrate after hMPV infection (histopathological score) compared to mice gestated in Eut or Hpx+T4 conditions (**Figure 4B**). Nevertheless, samples from Eut and Hpx+T4 infected mice were similar to their respective mocks (**Figures 4B, C**). On the other hand, the inflammatory process was located mainly in the peribronchial compartment. Accordingly, the peribronchiolar score also tended to be higher in the lungs of Hpx mice than in Eut or Hpx+T4 mice (**Figure 4C**). Given that we observed differences in the peribronchial compartment between Eut and Hpx gestated mice, a histological analysis was performed in lungs from uninfected experimental groups (**Figure S5A**). A trend of an increased number of terminal bronchioles in Hpx gestated mice was observed compared to Eut mice (see **Figure S5B**). Significantly, no statistical differences were observed when evaluating the expression of proinflammatory cytokines IL-6 and IFN-γ (**Figures S6A, B**) in the lung of the experimental groups. Although there is a trend toward higher basal IFNγ in uninfected Hpx mice compared to controls (**Figure S6B**). Besides, no differences were found in the expression of the anti-inflammatory cytokine IL-10 in the lung seven days after infection with hMPV (**Figure S6C**).

## Discussion

This is the first study that analyzes the pathological score and the immune response, against hMPV infection or other respiratory viral infection, in the adult mice gestated in Hpx. We observed that all experimental groups infected with hMPV reduced their weight and recovered their weight after seven days of infection as expected for mice gestated in euthyroidism (**Figures 1A, B**) (31). Even though, male offspring gestated in Hpx have high pathological scores after hMPV infection compared to Eut it was not significantly different (**Figure 1D** **);** instead, it was significantly different in female offspring gestated in Hpx compared to Eut and Hpx+T4 female (**Figure 1C**). These results are consistent with the histopathological analysis of the lung (**Figure 4**). This analysis showed that after seven days of infection, the offspring gestated in Hpx still have a higher histopathological and peribroncheoalveolar score than Eut. Our findings suggest that gestational Hpx induces a strong immune response in the offspring, especially in female offspring. In fact, the viral load in the lungs of female Hpx offspring was resolved by day seven; meanwhile, Hpx males did not reduce the viral load (**Figures 1E, F**). It has been described that a robust host immune response after a respiratory viral infection could induce strong inflammation damaging the surrounding tissue (32). This was observed in the histopathological score of Hpx (**Figure 4**) and could account for the increase in the clinical score (**Figure 1C**).

Accordingly, we observed that the female Hpx offspring have a higher proportion of CD8^+^ T cells than the Eut female groups (**Figures 2D** and **3**). Interestingly, previous results from our laboratory have demonstrated that hMPV triggers an aberrant T cells response, showing reduced expression of CD69, CD71 (transferrin receptor), and IL-2 secretion (33, 34). Here we observed a significant increment of CD8^+^ T cells with the intermediate marker CD71 compared to Eut and Hpx+T4 infected groups (**Figure 3B**). Similarly, we observed an increase in the proportion of CD8^+^ FASL^+^ and GRZB^+^ cells, indicating an increased effector capacity of these cells. These results suggest that the Hpx during gestation promotes a more robust adaptive immune response in female animals after hMPV infection. It has been reported that females presented higher CD8^+^ cytotoxic T cells after a viral respiratory infection than males (35, 36), and according to that, males are more susceptible to viral infection (35). Sex bias has been usually associated with the influence of sex hormones such as testosterone, progesterone and estrogens (36, 37). In this sense, gestation under Hpx could induce epigenetic changes that alter the production of sex hormones or the expression of its receptors, influencing the immune system´s performance against viral infections (38). Accordingly to our results is the report of adult female mice gestated under hypothyroidism condition infected with *Streptococcus pneumoniae* (20). In this report gestational hypothyroidism period protected against bacterial respiratory infection in adult female offspring (20).

Interestingly, the male offspring gestated in Hpx was the only experimental animal group that did not achieve viral clearance at day seven post-infection (**Figure 1F**). Since we only studied day seven after infection, the elevated viral load in the group of Hpx males could be due to alterations in the early innate response that was not detected in our experimental design. The experimental animal group that, during gestation, received MMI+T4 showed a phenotype like Eut animal group in the percentage of CD8^+^ T lymphocytes (**Figure 2D**). However, the results obtained for clinical score (**Figures 1C, D**), viral load (**Figures 1E, F**), and histopathological scores (**Figure 4**) of this experimental group were between Eut and Hpx offspring. This can be due to the MMI administration which significantly reduces the plasmatic levels of T_4_; the administration of T_4_ only partially recovers the plasmatic level of T_4_ (**Figure S1B**).

The evidence of this study supports the notion that thyroid hormone deficiency during gestation increases the immune response and inflammatory phenotype in the offspring, as was observed in the offspring induced with EAE (18). The clinical and histopathological scores of female Hpx offspring infected with hMPV were higher than those previously reported for female adult mice gestated under hypothyroidism infected with bacteria (20). The results are not contradictory because the robust immune response mounted against hMPV in the Hpx female group was more efficient in reducing viral load than males, as it was also observed in female mice gestated under hypothyroidism infected with bacteria. Thus, the increased clinical score of the Hpx group could result from tissue damage in the lungs induced by the robust inflammatory response. On the other hand, as the innate response against hMPV occurs early, we did not find differences in the neutrophil infiltrate on day seven (**Figures 2A, E**) nor in macrophages and dendritic cells infiltrates (**Figure S4**). Thus, it would be essential to study the response in the early time post-infection to establish whether differences in the neutrophil infiltrate promote the observed enhanced response of CD8^+^ T lymphocytes (36). Even though it is not clear the mechanism affected in the offspring gestated under thyroid hormone deficiency, it is clear that this condition affected the function of the immune system. Another aspect that could affect the outcome of the infection is lung development. We observed that at least adult mice gestated under the Hpx condition do not present important basal differences in the structures of the respiratory airway compared to Eut or Hpx+T4 groups (**Figure S5**). However, studies on the respiratory effects caused by deficient thyroid hormones have correlated it to a loss in cartilaginous tissue around the bronchi, reduced airway ventilation, and increased interstitial secretion (39, 40). On the other hand, increased permeability was reported in the lungs of mice gestated under hypothyroidism before the infection (20). These structural alterations could make children prone to recurrent respiratory infections (41).

Our results indicate that promoting a closer following of THs during pregnancy is essential. Remarkably, the fertile population has a high incidence of THs deficiency cases (hypothyroidism or hypothyroxinemia), mostly non-diagnosed during pregnancy (12). This work contributes to dimensioning the effects of gestational Hpx in gestation to offspring. Although the effect of maternal THs deficiency on the CNS development of the progeny has been reported, little information is available regarding their influence on the immune system function.

Moreover, this work is the first evidence that a THs deficiency during gestation can affect the immune response against viral infections in adult life. Significantly, our results could be underestimated since we studied adult progeny, and it is known that infants, young children, the elderly, and immunocompromised patients are the target population who develops more severe diseases caused by hMPV. Therefore, more pronounced differences in responses could perhaps be found when evaluating the performance of these risk groups gestated under Hpx condition.

The mechanisms that link the gestational THs deficiency with the alteration of the immune response against hMPV infections remains to be elucidated. However, it is known that prenatal disturbances have significant long-term programming effects on the offspring’s life. In this way, an overall assessment of the epigenetic, transcriptional, and/or translational profile of mice gestated in T_4_ deficiency would provide a broader picture and clues about which genetic pathways might be affected.

THs deficiency generates different consequences on the health of the progeny (42), and as we have discussed, the monitoring of transient Hpx in pregnant people is poorly established. Notably, the incidence of Hpx in pregnancy has been recently increased following the SARS-CoV-2 pandemic, probably due to the stress associated with confinement which influences the maternal tT_4_ (43).

Importantly, it is unknown the effect that pregnancy under thyroid hormone deficiency could have on the performance of the immune response against other respiratory viruses such as the SARS-CoV-2 virus. Infection with this virus is known to cause extensive lung damage due to the cytokine storm that produces runaway inflammation. In this sense, alterations induced in the lung structure (increased permeability), as well as high infiltrates of inflammatory immune cells could generate a greater susceptibility to developing a more severe COVID-19.

## Data availability statement

The raw data supporting the conclusions of this article will be made available by the authors, without undue reservation.

## Ethics statement

The animal study was reviewed and approved by Scientific Ethics Committee for the Care of Animals and the Environment of the Pontificia Universidad Católica de Chile.

## Author contributions

SF, MR, AF-F, D-RP, JS, MA-L, and FG-S carried out the experiments. SF wrote the manuscript with support from MR and AF-F. JV, PZ, and JR made the histopathological evaluations. EJ initial experimental work and conceptualization. CR and AK supervised the work and performed critical revision of the manuscript. All authors contributed to the article and approved the submitted version.

## Funding

The work was supported by CONICYT/FONDECYT no. 3150559, 1190830, Millennium Institute on Immunology and Immunotherapy, P09/016-f, ICN09_016, ACE 210015, Fondecyt #1191300, #1150862, #1150173, #1140010, #1161525., and ANID PAI SA77210051 (JAS) SCF is currently a CONICET postdoctoral fellow at IMBIO-SL Argentina.

## Conflict of interest

The authors declare that the research was conducted in the absence of any commercial or financial relationships that could be construed as a potential conflict of interest.

## Publisher’s note

All claims expressed in this article are solely those of the authors and do not necessarily represent those of their affiliated organizations, or those of the publisher, the editors and the reviewers. Any product that may be evaluated in this article, or claim that may be made by its manufacturer, is not guaranteed or endorsed by the publisher.

## Supplementary material

Supplementary Figure 1 MMI treatment induces a transitory gestational hypothyroxinemia condition. The experimental design of the induction of hypothyroxinemia (Hpx) and subsequent infection with hMPV is outlined. Pregnant female mice were treated from day 10 of pregnancy (P10) to day 15 of pregnancy (P15) with either MMI, MMI+T4 to revert the MMI phenotype or water as a control to generate three experimental groups in the offspring, Hpx, Hpx+T4, and Euthyroid (Eut), respectively **(A)**. The serum level of total T4 (tT4) **(B)**, TSH **(C)** and T3 **(D)** hormones were measured on the day (P15) of pregnant mice and T4 in the offspring gestated under Hpx, Hpx+T4, and Eut conditions at eight-to nine-week-old **(E)**. The averages of at least three independent serum measurements ± SEM are shown. One-way ANOVA analysis was performed **p < 0.01. MMI: methimazole; SEM: standard error of the mean; T4: 3,5,3’,5’-L-tetraiodothyronine, TSH: Thyroid-Stimulating Hormone.

Supplementary Figure 2 Gating strategy. The gating strategy used to differentiate cell populations in the lung of animals from the experimental groups is shown as following lymphoid cells, including B cells, CD4^+^ or CD8^+^ lymphocytes **(A)**. The gating strategy for identifying activated CD8 lymphoid cells is also shown **(B)**. On the other hand, the strategy to select neutrophil populations is graphed **(C)**.

Supplementary Figure 3 There is no change in anti-hMPV antibodies between Hpx-gestated mice and control mice. The graph shows the anti-hMPV IgG antibodies detected in the serum of mice seven days after infection with MPV or Mock. No significant differences are observed at this time. The data are expressed with the mean and the standard error of the values of each group.

Supplementary Figure 4 Pregnancy in hypothyroxinemia does not change the pulmonary infiltrate of myeloid cells seven days after infection with hMPV. Flow cytometry measured the proportion of interstitial **(A)** and alveolar macrophages **(B)** in the lung, as well as CD103+ **(C)** and CD11b+ **(D)** dendritic cells. Values are expressed as a percentage of the infiltrate of CD45+ cells. Two-way ANOVA and Tukey’s multiple comparisons test were performed.

Supplementary Figure 5 Bronchial structure in the progeny of Hpx mothers. Lung sections from non-infected Eut, Hpx, and Hpx+T_4_ are shown at 10X with H&E stain. Representative images of histological sections (600 µm) are shown. The black arrow shows the alveolus (AD) and terminal bronchioles (BT) for each group **(A)**. The number of terminal bronchioles for each treatment is graphed **(B)**. The averages from six to eight animals per group ± SEM are shown and analyzed by one-way ANOVA.

Supplementary Figure 6 Gestation under hypothyroxinemia did not alter the levels of inflammatory cytokines in the lung seven days after infection with hMPV. Using qPCR, the expression of the cytokines IL-6 **(A)**, IFN-γ **(B)** and IL-10 **(C)** in the lung on day seven post-infection was determined. Values were presented based on expression relative to the β-actin reference gene. Two-way ANOVA and Tukey’s multiple comparisons test were performed.
