# Supplementary figures and images for: Female offspring gestated in hypothyroxinemia and infected with human Metapneumovirus (hMPV) suffer a more severe infection and have a higher number of activated CD8+ T lymphocytes
Source: Front Immunol. 2022 Sep 8;13:966917. doi: 10.3389/fimmu.2022.966917 (PMC9494552; doi:10.3389/fimmu.2022.966917)

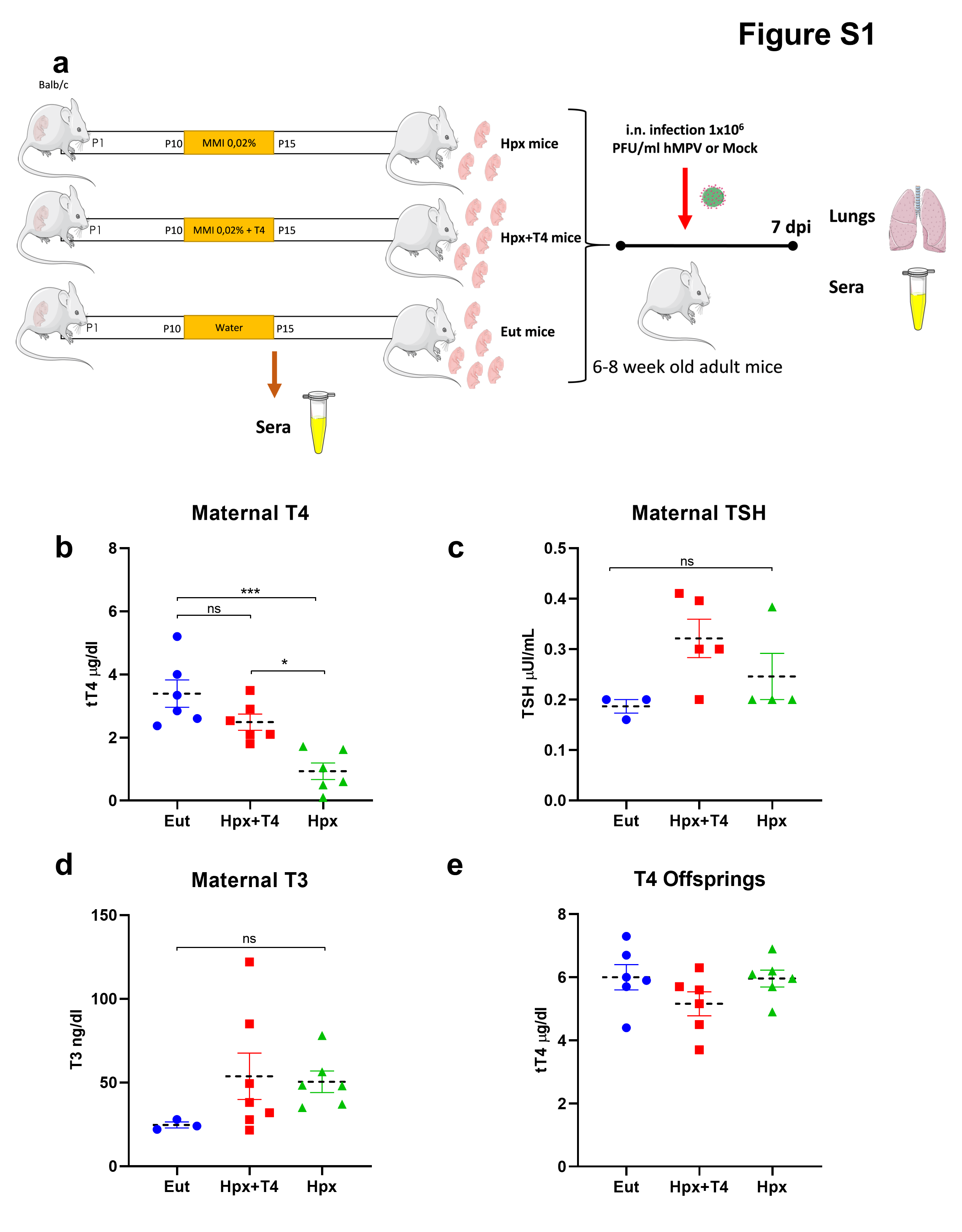

Supplement: Supplementary Figure 1 — MMI treatment induces a transitory gestational hypothyroxinemia condition. The experimental design of the induction of hypothyroxinemia (Hpx) and subsequent infection with hMPV is outlined. Pregnant female mice were treated from day 10 of pregnancy (P10) to day 15 of pregnancy (P15) with either MMI, MMI+T4 to revert the MMI phenotype or water as a control to generate three experimental groups in the offspring, Hpx, Hpx+T4, and Euthyroid (Eut), respectively (A). The serum level of total T4 (tT4) (B), TSH (C) and T3 (D) hormones were measured on the day (P15) of pregnant mice and T4 in the offspring gestated under Hpx, Hpx+T4, and Eut conditions at eight-to nine-week-old (E). The averages of at least three independent serum measurements ± SEM are shown. One-way ANOVA analysis was performed **p < 0.01. MMI: methimazole; SEM: standard error of the mean; T4: 3,5,3’,5’-L-tetraiodothyronine, TSH: Thyroid-Stimulating Hormone. [file Image_1.tif]

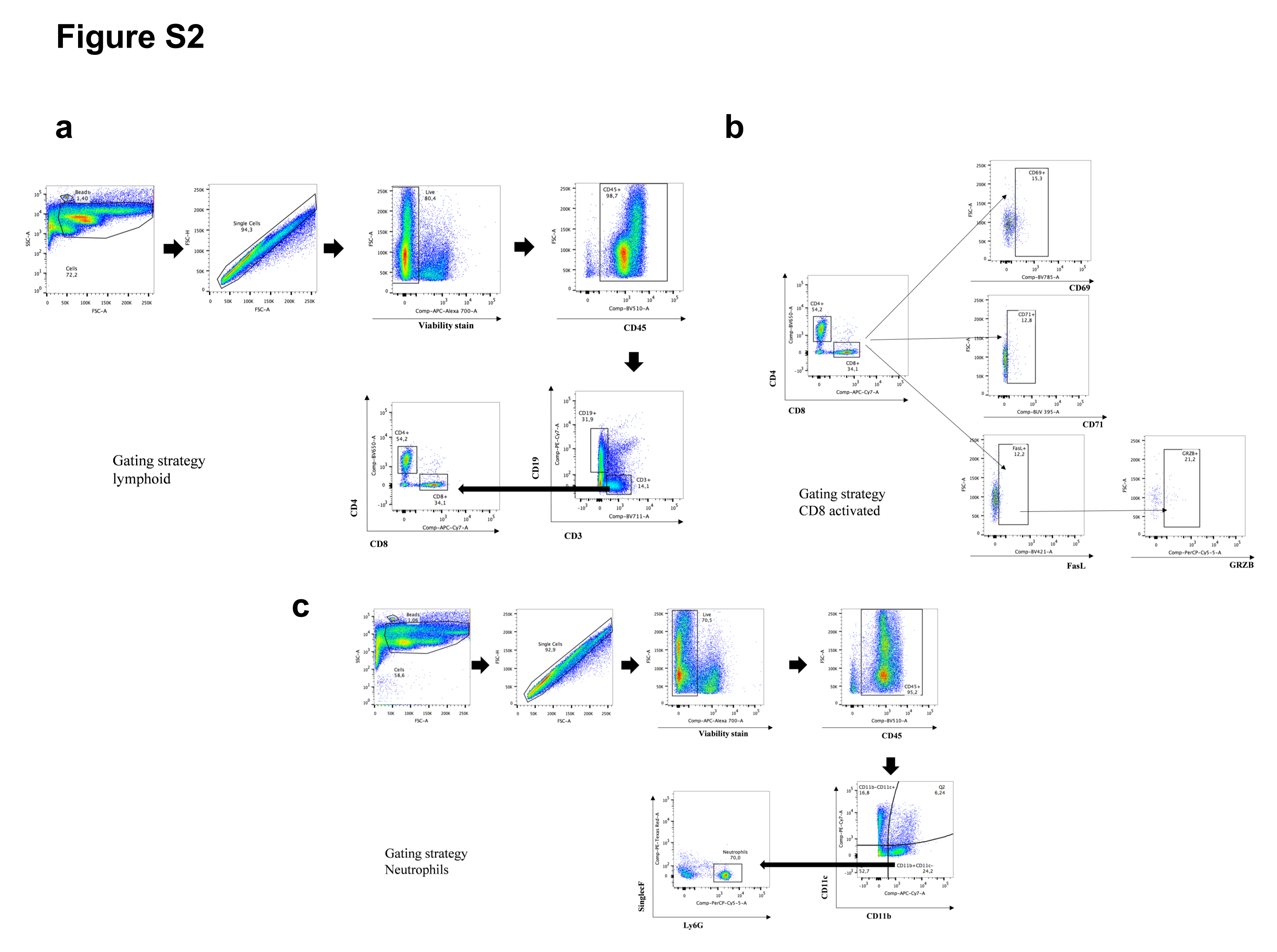

Supplement: Supplementary Figure 2 — Gating strategy. The gating strategy used to differentiate cell populations in the lung of animals from the experimental groups is shown as following lymphoid cells, including B cells, CD4+ or CD8+ lymphocytes (A). The gating strategy for identifying activated CD8 lymphoid cells is also shown (B). On the other hand, the strategy to select neutrophil populations is graphed (C). [file Image_2.tif]

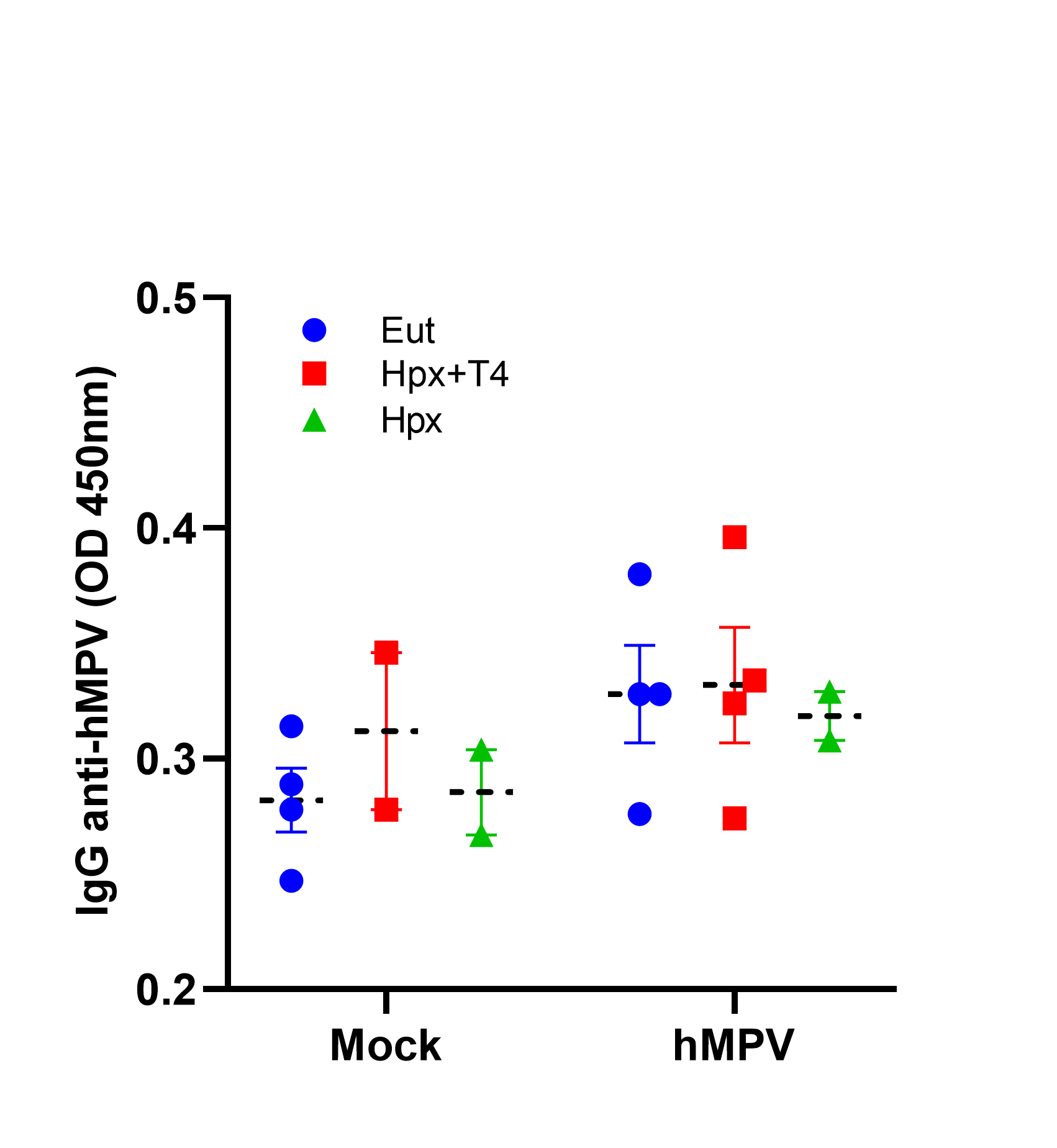

Supplement: Supplementary Figure 3 — There is no change in anti-hMPV antibodies between Hpx-gestated mice and control mice. The graph shows the anti-hMPV IgG antibodies detected in the serum of mice seven days after infection with MPV or Mock. No significant differences are observed at this time. The data are expressed with the mean and the standard error of the values of each group. [file Image_3.tif]

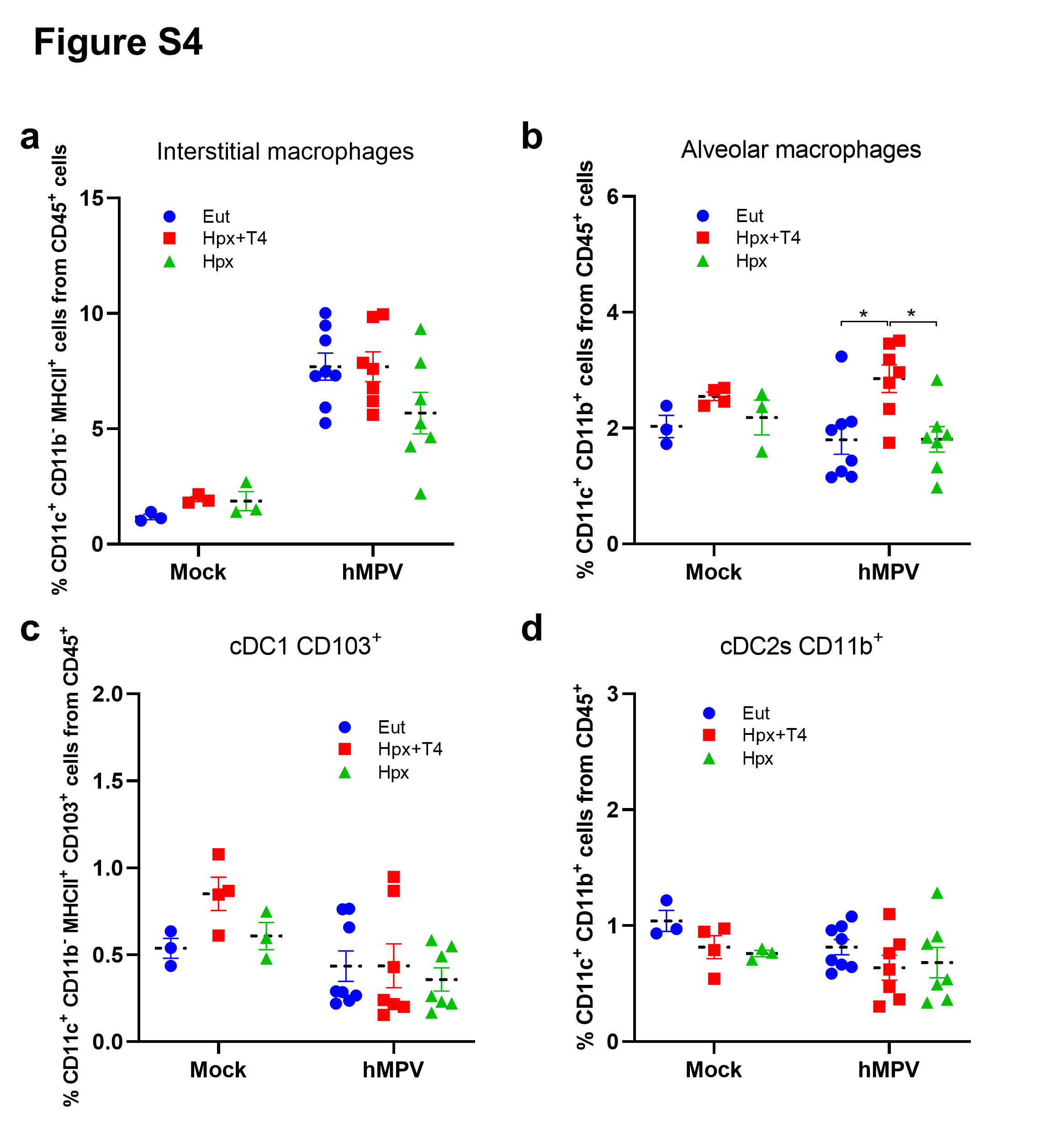

Supplement: Supplementary Figure 4 — Pregnancy in hypothyroxinemia does not change the pulmonary infiltrate of myeloid cells seven days after infection with hMPV. Flow cytometry measured the proportion of interstitial (A) and alveolar macrophages (B) in the lung, as well as CD103+ (C) and CD11b+ (D) dendritic cells. Values are expressed as a percentage of the infiltrate of CD45+ cells. Two-way ANOVA and Tukey’s multiple comparisons test were performed. [file Image_4.tif]

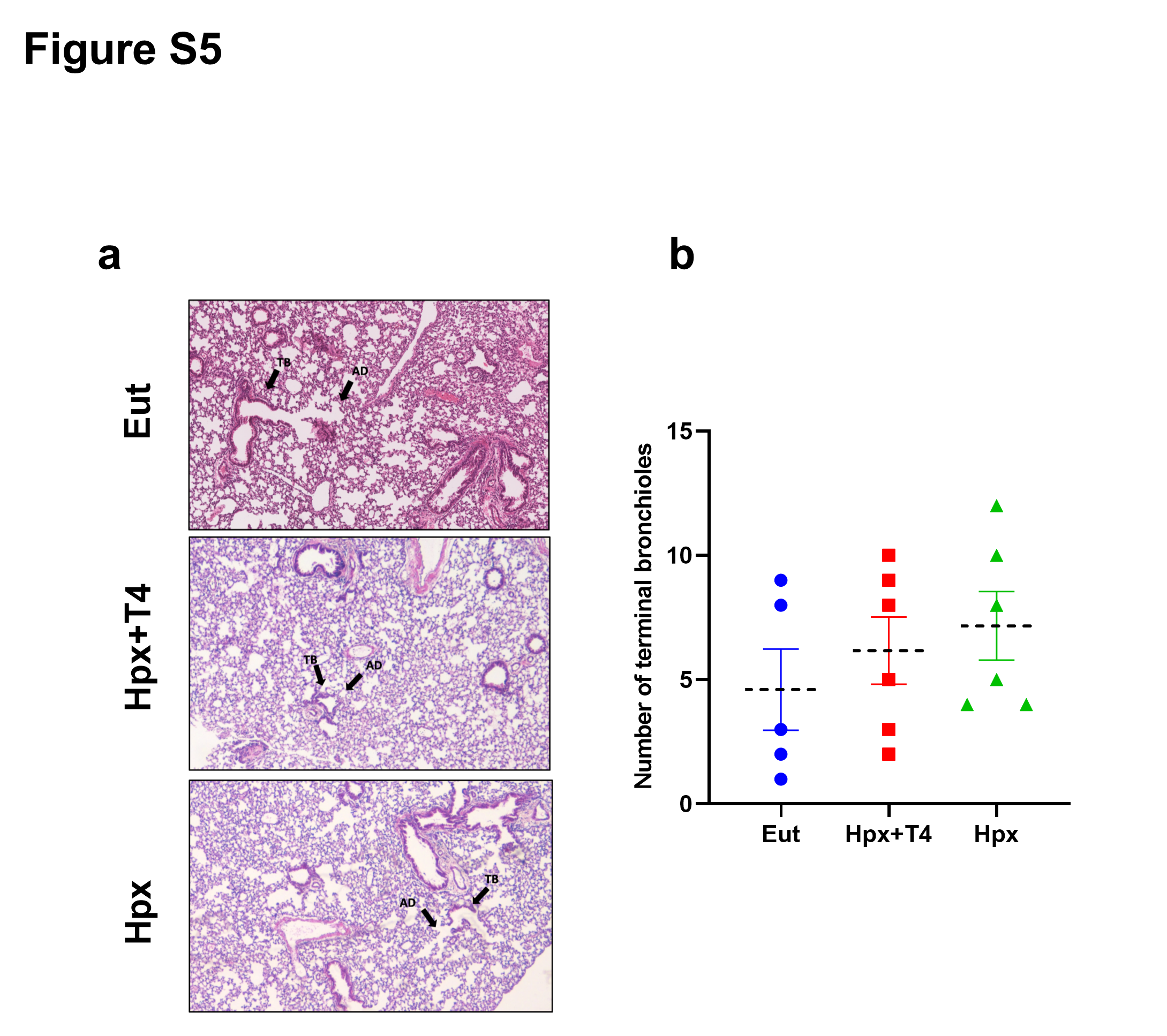

Supplement: Supplementary Figure 5 — Bronchial structure in the progeny of Hpx mothers. Lung sections from non-infected Eut, Hpx, and Hpx+T4 are shown at 10X with H&E stain. Representative images of histological sections (600 µm) are shown. The black arrow shows the alveolus (AD) and terminal bronchioles (BT) for each group (A). The number of terminal bronchioles for each treatment is graphed (B). The averages from six to eight animals per group ± SEM are shown and analyzed by one-way ANOVA. [file Image_5.tif]

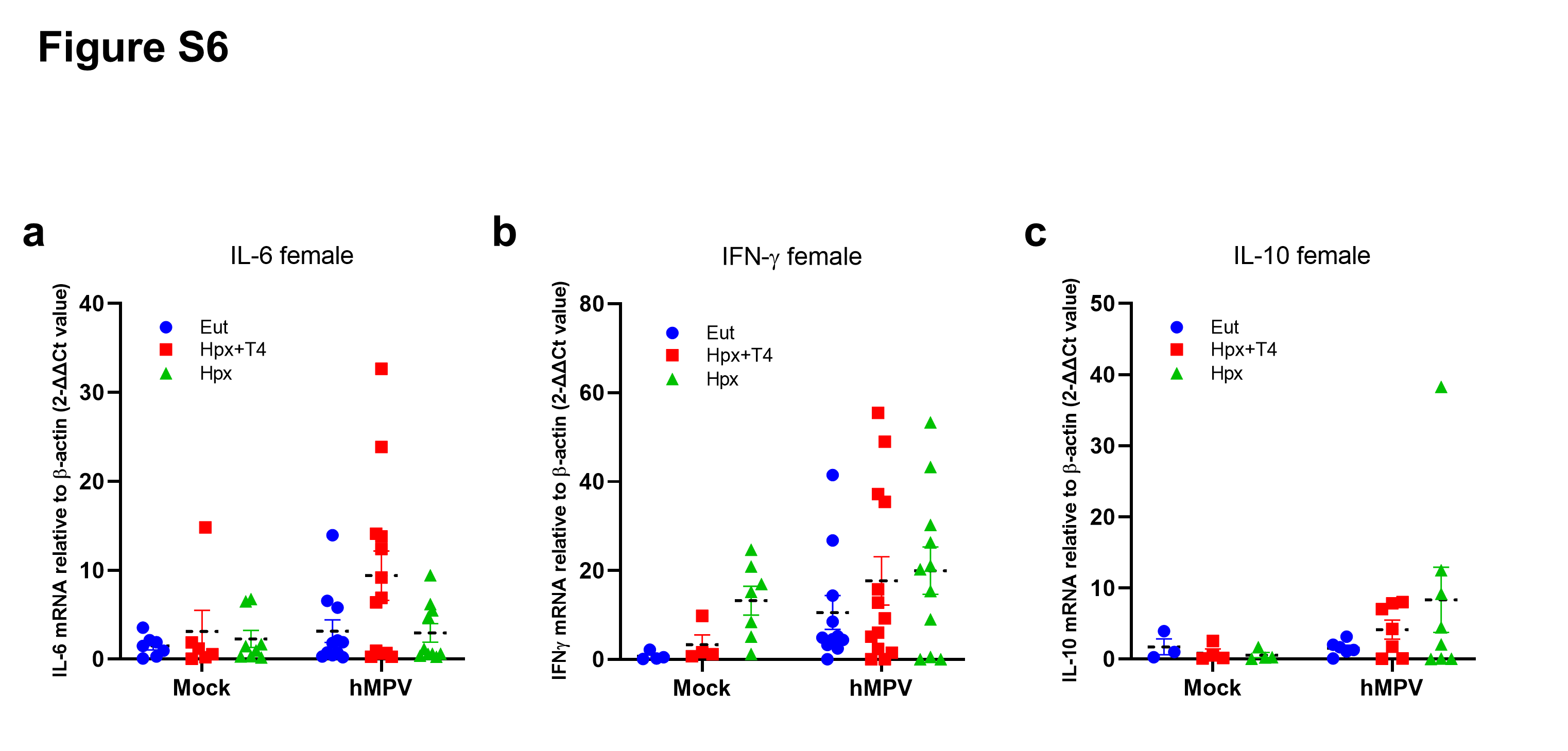

Supplement: Supplementary Figure 6 — Gestation under hypothyroxinemia did not alter the levels of inflammatory cytokines in the lung seven days after infection with hMPV. Using qPCR, the expression of the cytokines IL-6 (A), IFN-γ (B) and IL-10 (C) in the lung on day seven post-infection was determined. Values were presented based on expression relative to the β-actin reference gene. Two-way ANOVA and Tukey’s multiple comparisons test were performed. [file Image_6.tif]
